# Variation Is the Exploration of Possibilities

**DOI:** 10.3201/eid2012.AC2012

**Published:** 2014-12

**Authors:** Byron Breedlove

**Affiliations:** Centers for Disease Control and Prevention, Atlanta, Georgia, USA

**Keywords:** art science connection, emerging infectious diseases, invasive species, mutations, starlings, zoonotic infections, art and medicine, Fred Tomaselli, Starling, variation is the exploration of possibilities, about the cover

**Figure:**
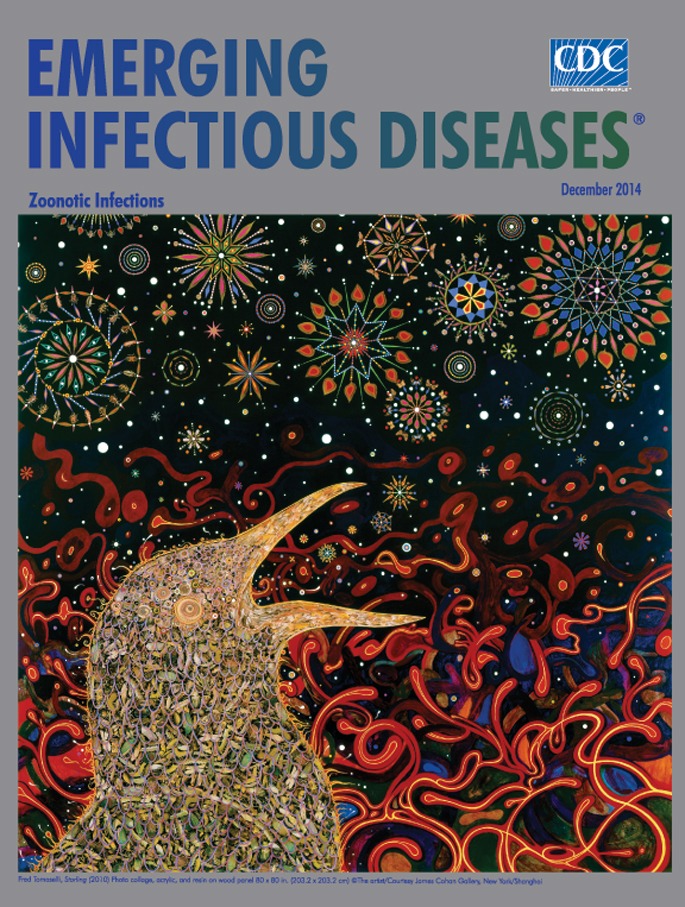
**Fred Tomaselli (b. 1956) Starling. 2010. Photo collage, acrylic, and resin on wood panel. 80 × 80 in/203.2 × 203.2 cm.** © The Artist. Courtesy James Cohan Gallery, New York/Shanghai

Born in 1956, American artist Fred Tomaselli grew up in Orange, California, and in 1982, he graduated from California State University, Fullerton, with a degree in painting and drawing. In the early 1980s, Tomaselli became established in the “Downtown L.A.” art, music, and drug countercultures. Since 1985, he has lived and worked in Brooklyn, New York.

He is best known for his visually stunning hybrid creations—part painting and part mosaic—and for his series of collages based on front-page articles from *The New York Times*. His works have been exhibited widely throughout the world and are in collections at many prominent museums.

Tomaselli collects and incorporates unusual materials into his works, including pharmaceutical and street drugs and myriad images of plants, birds, and body parts cut from catalogs and magazines, suspending them in layers of clear epoxy resin. If bugs, leaves, and other debris become trapped in the sticky resin, Tomaselli keeps them. He acknowledges the influence of Eastern and Western decorative traditions such as quilts, tapestries, and mosaics, art forms that feature details and repetition. Tomaselli considers his approach to art as a way of reorganizing and reframing information. He once explained to an interviewer, “When I combine these little chunks of information, it’s not unlike the way nature stacks up genes to build everything from viruses to humans. I tend to see each small bit like an individual cell, a piece of binary code, or a strand of DNA that accumulates, accrues, and grows into my images.”

Tomaselli wants viewers to lose themselves in his art, even to the point of experiencing the sort of confusion and exaltation attributed to Stendhal syndrome. He describes his art as being “about artificial immersive environments, about escapism,” themes spawned by his upbringing in suburban California, where amusement parks, shopping malls, and recreational drugs were pervasive.

The artist created this month’s dazzling cover image, *Starling*, for an exhibit at the Brooklyn Museum. The starling’s head juts from the bottom left amidst searing fluid-like ribbons of red, blue, and yellow. Various insects, perhaps food the bird has eaten, comprise its neck and stomach. This stylized starling may be launching into one of its species’ complex soliloquies or devouring figs from the trees growing in the artist’s yard. Radiant, precise pulsing kaleidoscopic patterns float across the top third of the painting. The bird’s head provides a reference point, but exploring the array of swirling colors, exploding shapes, dots, and specks proves irresistible. The juxtaposition of colorful microscopic and celestial images contrasted against a black background recurs in much of Tomaselli’s art.

Starlings were first introduced into the United States in 1890 when Eugene Schieffelin released 60 of them in New York City’s Central Park, not far from the Brooklyn Museum that now houses Tomaselli’s *Starling*. Schieffelin, who belonged to the American Acclimatization Society, wanted to introduce each species of bird mentioned in the Shakespeare’s works into the United States. (Shakespeare mentioned a starling in *Henry IV*, Part 1, Act 1, Scene 3, wherein Hotspur seeks revenge on King Henry, who refuses to pay the ransom for Hotspur’s brother-in-law Mortimer: “Nay / I’ll have a starling shall be taught to speak / Nothing but ‘Mortimer,’ and give him/ To keep his anger still in motion.”)

An estimated 200 million of these iridescent interlopers, recognized by their purple and green chests and throats and their rasping, screeching songs, are entrenched across the United States (invasive starlings are also found in Canada, South Africa, New Zealand, and Australia). A *New York Times* article written a century after their importation notes that the starling “has distinguished itself as one of the costliest and most noxious birds on our continent.” Starlings roost in massive colonies, displace native species of birds, pose problems for air travel, damage crops and fruit trees, and help spread diseases, offsetting any benefits they add by eating insects.

Starlings and other bird species could potentially harbor harmful agents, including those that cause influenza, histoplasmosis, cryptococcosis, and West Nile encephalitis in humans. When present in massive numbers or under the right circumstances, such wild bird populations may be more than a nuisance and possibly pose health threats. Some pathogens hosted by bird species are capable of reorganizing and reframing their genetic information. In his recently published book *Spillover: Animal Infections and the Next Human Pandemic*, David Quammen used the phrase “variation is the exploration of possibilities” when discussing mutations in viruses—which also sounds like Tomaselli’s approach to creating his multifaceted collages.
